# The association mental health of adolescents with economic impact during the COVID-19 pandemic: a 2020 Korean nationally representative survey

**DOI:** 10.1186/s12889-023-15808-3

**Published:** 2023-05-11

**Authors:** Hanul Park, Kang-Sook Lee

**Affiliations:** 1 Korea Disease Control and Prevention Agency (KDCA), 187 Osongsaengmyeong 2-ro, Cheongju-si, Chungcheongbuk-do 28159 Republic of Korea; 2 Department of Preventive Medicine, College of Medicine, The Catholic University of Korea, 222 Banpo-daero, Seocho-gu, Seoul, 06591 Republic of Korea

**Keywords:** COVID-19 pandemic, Economic impact, Adolescent health, Mental health, Health equality

## Abstract

**Background:**

COVID-19 has affected innumerable aspects of life, including education, economy, and religion. Economic problems and inequality are associated with poor mental health in adolescents. This study aimed to identify the relationship between economic damage to families due to COVID-19 and various mental health problems in Korean adolescents and to evaluate the risk factors of mental health.

**Methods:**

In total, 54,948 Korean adolescent students from 398 middle and 395 high schools were surveyed between August and November 2020. Complex sample logistic regression was performed to calculate odds ratios (ORs) and 95% confidence intervals (CI) for depression and suicidal ideation, respectively. A generalized linear model analysis was used to examine the association between mental health (unhappiness, loneliness, and stress) and the economic impact of COVID-19. Analyses were adjusted for age, gender, school grade, perceived academic achievement, perceived family economic status, and economic support.

**Results:**

The ORs of depression (OR = 1.77, 95% CI:1.57–2.00), suicidal ideation (OR = 2.14, 95% CI:1.84–2.50), unhappiness (OR = 1.51 95% CI 1.42–1.60) and lonely (OR = 1.38 95% CI 1.27–1.49) for the low level of perceived family economic status was higher compared to middle level. Adolescents who experienced economic deterioration in their households as COVID-19 showed a higher risk of depression (OR = 1.42, 95% CI:1.35–1.49), suicide ideation (OR = 1.36, 95% CI:1.28–1.44), unhappiness (OR = 2.23 95% CI 2.19–2.27), lonely (OR = 1.20 95% CI 1.17–1.22), and stress (OR = 1.14 95% CI 1.12–1.16) than those who did not.

**Conclusions:**

The findings revealed an association between the decline in household economic status due to COVID-19 and mental health problems, such as stress, loneliness, suicidal ideation, depression, and unhappiness.

**Supplementary Information:**

The online version contains supplementary material available at 10.1186/s12889-023-15808-3.

## Background

According to the World Health Organization (WHO) [1], as of January 18, 2023, the coronavirus disease (COVID-19) pandemic has resulted in over 6.7 million deaths and approximately 663 million confirmed cases worldwide. COVID-19 has affected innumerable aspects of life, including education, economy, and religion [2]. Unemployment increased substantially and inequality in the economic system deepened [3]. Research has shown that economic problems and inequality are related to poor mental health in adolescents [4]. Another study has shown a relationship between the economic impact of COVID-19 on households and increased suicide attempts in adolescents [5]. Children from families with low socioeconomic status are unlikely to receive psychosocial resources and support from their families, education, or peer environments [6]. A systematic literature review examining the relationship between COVID-19 and mental health of children and adolescents showed that fear, depression, and anxiety were higher after the pandemic than before the pandemic [7]. Additionally, the relationship between loneliness and the stay-at-home orders issued by the state to mitigate the COVID-19 pandemic was confirmed. Loneliness has been reported to be a serious public health issue in the fight against the infectious disease [8]. Previous studies have reported that the association between policies such as social distancing and the prevalence of mental health problems varies with age [9]. At the peak of the COVID-19 pandemic, stress and emotional crises in adolescents caused anxiety and depression [10].

As of April 3, 2023, South Korea had a total number of 30,843,900 confirmed cases and a death toll of 34,281 due to COVID-19. Of these, 3,839,761 (12.45%) confirmed cases and twenty-three (0.07%) deaths occurred among teenagers aged 10–19 years [11]. The Ministry of Education of the Republic of Korea announced a policy to conduct online classes during the pandemic and designated dates on which each grade could attend school to minimize crowding in educational institutions. Studies indicate that COVID-19 rarely affects children and adolescents directly [12]; however, research has shown that there may be direct or indirect effects on mental and physical health due to isolation resulting from school closure, stay-at-home, and limited peer interactions [13]. In addition, adolescence is particularly sensitive to social stimulation, and the need for peer interaction increases [14]; however, social distancing has fundamentally reduced the opportunity for adolescents to make direct social contact outside of home. During the COVID-19 pandemic, non-routine experiences in adolescents were the main stressor [10]. Previous studies have also highlighted the urgent need to protect children and adolescents from the potential side effects of the COVID-19 pandemic [15]. Confirming the long-term negative effects of social isolation caused by COVID-19 on human physical and mental health is a challenge [2].

Therefore, it is important to identify and evaluate risk factors related to mental health problems in adolescents during the COVID-19 pandemic using data representing Korean youth. The purpose of this study was to identify the relationship between economic damage to families due to COVID-19 and various mental health problems in Korean adolescents and to evaluate the risk factors of mental health.

## Methods

### Sample and data collection

The current study was based on the 16th 2020 Korean Youth Risk Behavior Web-based Survey (KYRBS), which is a nationally representative survey of Korean youth health status, including smoking and drinking status, physical activity, obesity, nutrition, and mental health [16]. The purpose of the KYRBS is to collect data to plan and evaluate health promotion projects for the youth. In addition, these data have been used by international organizations such as the WHO to compare adolescent health conditions across countries.

This survey is conducted annually by The Korea Disease Control and Prevention Agency (KDCA) and is an anonymous online survey that asks middle and high school students to self-report. The teacher led the sample class to an Internet-enabled school computer room, and randomly assigned each person to a computer. Some schools had difficulty conducting the survey in the computer room due to COVID-19, and instead used mobile devices (tablet PCs and smartphones) in the classroom under the supervision of the teacher. The entire investigation process lasted 45–50 min. After completion, the teacher recorded the number of students who participated and those who were unable to. This information was then registered online at the time of the investigation. These data were used for the weight calculation. The KYRBS consisted of 103 questions and 93 indicators and was collected from August to November 2020. For a representative sample of Korean adolescents, complex sampling was designed through stratification, cluster, and multi-level sampling, and weight was assigned to each sample to represent the Korean adolescents. The calculation of biased results was controlled through the composite sample design, and the value was calculated using the weighted average. The weights for each variable in our study were reflected in the original data provided by the KDCA. The weights were calculated by multiplying the values obtained from the reciprocal of the extraction rate and the reciprocal of the response rate, and then multiplying that result by the weight post-correction rate. The survey was conducted with 57,925 students in 800 schools, 400 middle schools, and 400 high schools, but a total of 793 schools (398 middle schools and 395 high schools), 54,948 due to the overburden of teachers and the inability to use the computer lab due to COVID-19. The participation rate, based on the number of students, was 94.9% [17]. The Institutional Review Board of the Catholic University of Korea reviewed and approved the study design (IRB approval number: MC22ZASI0021).

### Measures

#### Economic variables

In our study, the economic variables were perceived family economic status, economic support, and the economic impact of COVID-19. Perceived family economic status was measured using the question, “What is the economic status of your family?” with response options: “High,” “Middle-High,” “Middle,” “Middle-Low,” and “Low.” Economic support was assessed using the following question: “In the last 12 months, have you received financial help from non-family members or institutions due to poor family economic status?”. Responses options were “Yes” and “No.” The economic impact of COVID-19 was assessed using the following question: “Do you think the economic status of your family has become more difficult than before due to COVID-19?”. Response options were “Extremely likely,” “Somewhat likely,” “Not too likely” and “Not likely at all.” We categorized these responses into two groups: “Yes” and “No.”

#### Mental health

Depression and suicidal ideation were measured using the following questions: “Have you experienced sadness or despair to the point that you stopped your daily routine for two weeks?” and “In the past 12 months, have you ever thought of committing suicide?” The response options were: “Yes” or “No.” Unhappiness was measured with following question: “How happy do you usually feel?” The response options were: (1) I am very happy, (2) I am a bit happy, (3) It is normal, (4) I am a little unhappy, and (5) I am very unhappy. Loneliness was measured with the following question: “In the last 12 months, how often have you felt lonely?” The response options were: (1) I did not feel lonely at all, (2) I almost never felt lonely, (3) I sometimes felt lonely, (4) I often felt lonely, and (5) I always felt lonely. Stress was measured using the following question: “How much stress do you usually feel?” The response options were: (1) I do not feel it at all, (2) I do not feel it much, (3) I feel it a little, (4) I feel it a lot, and (5) I feel it very much.

### Statistical analysis

Based on the analytical guidelines of the KDCA, all analyses were conducted using IBM SPSS Statistics 28.0. Complex sample weights were applied to reflect nationally representative samples. We identified the general characteristics and economic impacts of COVID-19 using descriptive statistics. Chi-square tests were conducted to compare differences between groups. Complex sample logistic regression was performed to calculate odds ratios (ORs) and 95% confidence intervals (CI) for depression and suicidal ideation, respectively. A generalized linear model analysis was used to examine the association between mental health (unhappiness, loneliness, and stress) and the economic impact of COVID-19. Analyses were adjusted for age, gender, school grade, perceived academic achievement, perceived family economic status, and economic support. The significance level was set at *P* < 0.05.

## Results

Table 1 presents adolescents’ the demographic characteristics and economic impact of COVID-19 on the adolescents in our study. A total of 54,948 subjects participated in our study, of whom 26,595 (48.4%) were girls and 28,353 (51.6%) were boys. The number of participants was 10,005 (18.2%) in first year in middle school, and the number of participants decreased as the school grades increased. Of the participants, 16,585 (30.2%) reported moderate academic achievement. The perceived family economic status of the participants was as follows: 26,397 (47.5%) middle, 15,300 (28.6%) middle-high, and 6,039 (11.2%) high. In the last 12 months, 5,563 (10.1%) participants received economic support for family economic difficulties. A total of 16,839 (30.6%) participants reported that the economic status of their family had become more difficult due to COVID-19.

**Table 1:** Demographic characteristics and economic impact of COVID-19 in the KYRBS 2020 adolescents

**Variables**	**Participants** **N(%)**
**Total**	54,948
**Age (median)**	15(14–17)
**Gender**	
Boys	28,353(51.6)
Girls	26,595(48.4)
**School grade (14-19 years)**	
Middle school 1^st^	10,005(18.2)
Middle school 2^nd^	9,564(17.1)
Middle school 3^rd^	9,392(17.1)
High school 1^st^	8,907(16.2)
High school 2^nd^	8,907(16.2)
High school 3^rd^	8,173(14.9)
**Perceived academic achievement (in the last 12 months)**	
High	6,736(12.3)
Middle-High	13,410(24.4)
Middle	16,585(30.2)
Middle-Low	12,684(23.1)
Low	5,533(10.1)
**Perceived family economic status**	
High	6,039(11.2)
Middle-High	15,300(28.6)
Middle	26,397(47.5)
Middle-Low	5,937(10.4)
Low	1,275(2.2)
**Economic support (in the last 12 months)**	
No	49,385(89.9)
Yes	5,563(10.1)
**Economic impact of COVID-19**	
No	38,109(69.4)
Yes	16,839(30.6)

**Fig. 1:**
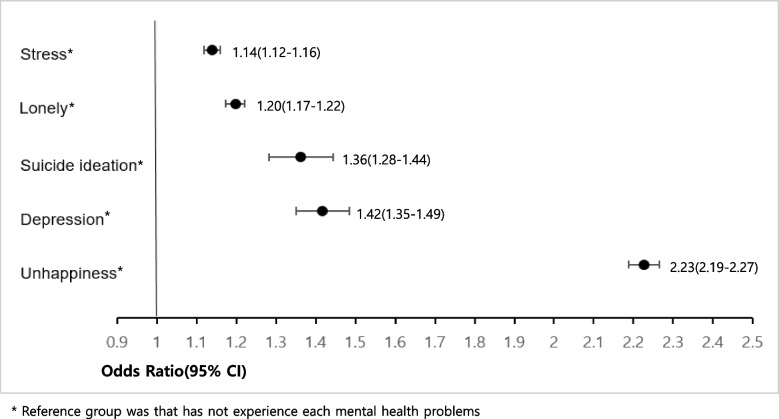
The adjusted odds ratio of mental health by economic impact of COVID-19 in the KYRBS 2020 adolescents (*n* = 54,948) in Korea

Table 2 shows ORs of depression and suicidal ideation which were adjusted for age, gender, school grade, perceived academic achievement, perceived family economic status, economic support, and economic impact of COVID-19. Compared with boys, girls had a higher risk of depression (OR = 1.80, 95% CI: 1.72–1.88) and suicidal ideation (OR = 1.88, 95% CI: 1.76–2.00). Compared to those in first year in middle school, second-year high school students had a higher risk of depression (OR = 1.37, 95% CI: 1.14–1.65) and high school third-year students also had a higher risk of suicidal ideation (OR = 1.64, 95% CI: 1.20–2.24). A low level of perceived academic achievement was associated with a higher risk of depression (OR = 1.61, 95% CI: 1.51–1.73) and suicidal ideation (OR = 1.78, 95% CI: 1.62–1.95) compared to a middle level. The depression (OR = 1.77, 95% CI: 1.57–2.00) and suicide risk (OR = 2.14, 95% CI: 1.84–2.50) of a low level of perceived family economic status was higher compared to a middle level. Adolescents of family that had received financial support in the past 12 months had a higher risk of depression (OR = 1.59, 95% CI: 1.39–1.82) and suicidal ideation (OR = 1.59, 95% CI: 1.39–1.82) than those who did not. Adolescents who experienced economic deterioration in their households as COVID-19 showed a higher risk of depression (OR = 1.42, 95% CI: 1.35–1.49) and suicidal ideation (OR = 1.36, 95% CI: 1.28–1.44) than those who did not (Fig. 1).

**Table 2:** Multivariate logistic regression analysis of factors influencing depression and suicide ideation in the KYRBS 2020 adolescents (*n* = 54,948) in Korea

**Variables**	**Depression**		**Suicide ideation**	
	aOR^*^	**(95%CI)**	aOR^*^	**(95%CI)**
**Gender**				
Girls vs Boy	1.80	(1.72–1.88)	1.88	(1.76–2.00)
**School grade (15-19 years vs 14 years)**				
Middle school 2^nd^ vs Middle school 1^st^	1.14	(1.04–1.25)	1.26	(1.11–1.42)
Middle school 3^rd^ vs Middle school 1^st^	1.27	(1.13–1.42)	1.36	(1.16–1.59)
High school 1^st^ vs Middle school 1^st^	1.22	(1.05–1.42)	1.29	(1.05–1.58)
High school 2^nd^ vs Middle school 1^st^	1.37	(1.14–1.65)	1.62	(1.25–2.09)
High school 3^rd^ vs Middle school 1^st^	1.18	(1.18–1.84)	1.64	(1.20–2.24)
**Perceived academic achievement (in the last 12 months)**				
High vs Middle	0.89	(0.83–0.96)	1.14	(1.03–1.26)
Middle-High vs Middle	0.94	(0.89–0.99)	1.06	(0.98–1.14)
Middle-Low vs Middle	1.20	(1.13–1.26)	1.25	(1.15–1.36)
Low vs Middle	1.61	(1.51–1.73)	1.78	(1.62–1.95)
**Perceived family economic status**				
High vs Middle	1.11	(1.04–1.20)	0.97	(0.87–1.08)
Middle-High vs Middle	1.12	(1.07–1.17)	1.09	(1.02–1.17)
Middle-Low vs Middle	1.30	(1.22–1.39)	1.55	(1.42–1.70)
Low vs Middle	1.77	(1.57–2.00)	2.14	(1.84–2.50)
**Economic support (in the last 12 months)**				
Yes vs No	1.16	(1.09–1.23)	1.21	(1.11–1.33)
**Economic impact of COVID-19**				
Yes vs No	1.42	(1.35–1.49)	1.36	(1.28–1.44)

^*^ Adjusted for age, gender, school grade, perceived academic achievement, perceived family economic status, economic support, economic impact of COVID-19

Table 3 presents the results of the multivariate logistic regression analysis. We adjusted ORs and 95% confidence intervals for unhappiness, loneliness, and stress related to the general characteristics and economic impact of COVID-19. Girls were more likely to feel unhappiness (OR = 1.21 95% CI: 1.19–1.23), loneliness, (OR = 1.46 95% CI: 1.43–1.49) and stress (OR = 1.36 95% CI: 1.34–1.39) than boys. High school third-year students were more likely to feel unhappiness (OR = 1.23 95% CI: 1.12–1.36) and stress (OR = 1.27 95% CI: 1.15–1.40) than those in first-year in middle school. Adolescents with a low level of perceived academic achievement had a higher risk of unhappiness (OR = 1.31 95% CI: 1.26–1.36), and higher risk of loneliness (OR = 1.17 95% CI: 1.13–1.22) than those who reported a middle level. Those who reported a low perceived economic status were more likely to feel unhappiness (OR = 1.51 95% CI: 1.42–1.60) and loneliness (OR = 1.38 95% CI: 1.27–1.49) than those who reported a middle status. Adolescents who had received financial support over the past 12 months were more likely to feel loneliness (OR = 2.59 95% CI: 2.50–2.67) than those who did not. Adolescents who experienced economic deterioration due to COVID-19 had a higher risk of unhappiness (OR = 2.23 95% CI: 2.19–2.27), loneliness (OR = 1.20 95% CI: 1.17–1.22), and stress (OR = 1.14 95% CI: 1.12–1.16) than those who did not (Fig. 1).

**Table 3:** Generalized linear model analysis of factors influencing unhappiness, lonely and perceived stress in the KYRBS 2020 adolescents (*n* = 54,948) in Korea

**Variables**	**Unhappiness**					**Lonely**					**Stress**				
	***β***	**SE**	aOR^*^	**95%CI**	***P***	***β***	**SE**	aOR^*^	**95%CI**	***P***	***β***	**SE**	aOR^*^	**95%CI**	***P***
**Gender**															
Girls vs Boy	0.19	0.01	1.21	1.19–1.23	< 0.001	0.38	0.01	1.46	1.43–1.49	< 0.001	0.31	0.01	1.36	1.34–1.39	< 0.001
**School grade (14-19 years)**															
Middle school 2^nd^ vs Middle school 1^st^	0.08	0.02	1.08	1.05–1.12	< 0.001	0.07	0.02	1.07	1.04–1.11	< 0.001	0.05	0.02	1.05	1.01–1.09	0.01
Middle school 3^rd^ vs Middle school 1^st^	0.11	0.02	1.12	1.07–1.17	< 0.001	0.11	0.03	1.12	1.06–1.17	< 0.001	0.09	0.02	1.09	1.04–1.15	< 0.001
High school 1^st^ vs Middle school 1^st^	0.01	0.03	1.01	0.95–1.08	0.004	0.15	0.03	1.16	1.09–1.24	< 0.001	0.09	0.03	1.09	1.03–1.16	0.007
High school 2^nd^ vs Middle school 1^st^	0.17	0.04	1.19	1.10–1.28	< 0.001	0.17	0.04	1.19	1.09–1.29	< 0.001	0.17	0.04	1.19	1.10–1.28	< 0.001
High school 3^rd^ vs Middle school 1^st^	0.21	0.05	1.23	1.12–1.36	< 0.001	0.15	0.05	1.16	1.05–1.29	0.006	0.24	0.05	1.27	1.15–1.40	< 0.001
**Perceived academic achievement**															
High vs Middle	-0.10	0.02	0.90	0.87–0.94	< 0.001	0.05	0.02	1.05	1.01–1.09	0.008	-0.01	0.02	0.99	0.95–1.03	0.76
Middle-High vs Middle	-0.04	0.01	0.96	0.94–0.98	< 0.001	0.04	0.01	1.04	1.02–1.06	0.002	0.01	0.01	1.01	0.99–1.03	0.69
Middle-Low vs Middle	0.10	0.01	1.11	1.08–1.13	< 0.001	0.10	0.01	1.11	1.08–1.13	< 0.001	0.08	0.01	1.08	1.06–1.10	< 0.001
Low vs Middle	0.27	0.02	1.31	1.26–1.36	< 0.001	0.16	0.02	1.17	1.13–1.22	< 0.001	-0.17	0.02	0.84	0.81–0.88	< 0.001
**Perceived family economic status**															
High vs Middle	-0.31	0.01	0.73	0.72–0.75	< 0.001	-0.17	0.02	0.84	0.81–0.88	< 0.001	0.13	0.02	1.14	1.10–1.18	< 0.001
Middle-High vs Middle	-0.13	0.01	0.88	0.86–0.90	< 0.001	0.02	0.01	1.02	1.00–1.04	0.062	-0.004	0.01	1.00	0.98–1.02	0.69
Middle-Low vs Middle	0.25	0.02	1.28	1.23–1.34	< 0.001	0.25	0.02	1.28	1.23–1.49	< 0.001	-0.18	0.01	0.84	0.82–0.85	< 0.001
Low vs Middle	0.41	0.03	1.51	1.42–1.60	< 0.001	0.32	0.04	1.38	1.27–1.49	< 0.001	-0.31	0.03	0.73	0.69–0.78	< 0.001
**Economic support (in the last 12 months)**															
Yes vs No	-0.52	0.02	0.59	0.58–0.61	< 0.001	0.95	0.02	2.59	2.50–2.67	< 0.001	-0.03	0.014	0.97	0.94–1.00	0.033
**Economic impact of COVID-19**															
Yes vs No	0.80	0.01	2.23	2.19–2.27	< 0.001	0.18	0.01	1.20	1.17–1.22	< 0.001	0.13	0.01	1.14	1.12–1.16	< 0.001

^*^ Adjusted for age, gender, school grade, perceived academic achievement, perceived family economic status, economic support, economic impact of COVID-19

## Discussion

Our study identified the connection between economic damage to households due to COVID-19 and mental health problems in adolescents during the pandemic and evaluated the risk factors for mental health problems. Our study found that mental health issues were more severe in girls than in boys. Previous studies have reported that the prevalence of mental health problems is higher in women than in men [18, 19], and have discussed genetics, hormones, endocrine stress reactivity, and personality factors to explain this phenomenon [18]. Major global events, such as COVID-19, may be related to endocrine stress reactivity.

Our results showed that, during the COVID-19 pandemic, girls experienced depression 1.8 times more often than boys. A prior study investigating the prevalence of depression in all age groups in Korea before the pandemic found that it was slightly higher among female adolescents than male adolescents [20], a finding similar to ours. According to a study that used 2020 data to compare the prevalence of depression among Korean adults before and after the COVID-19 pandemic, it increased only in men [21]. COVID-19 has been prevalent worldwide for two years, and more data must be collected and evaluated to understand the changes in mental health by gender due to the pandemic.

Compared to first-year middle school students (aged 14 years), our study found a greater prevalence of mental health problems in students of other grades (ages 15–19 years). Third-year high school students (aged 19 years) showed higher levels of suicidal ideation, unhappiness, and stress. A previous study reported that Korean adolescents experience stress and mental health problems because of concerns related to academic performance, which tend to worsen in high school [22]. In a study conducted before the COVID-19 outbreak, the Korea Institute for Health and Social Affairs confirmed that the prevalence of poor mental health in adolescents increased as they advanced through school years [23]. During the COVID-19 pandemic, adolescents have attended online classes, faced limitations in private tutoring while isolated, and lost formal educational opportunities. The mental health of third-year high school students (aged 19 years) during the COVID-19 pandemic was significantly worse than that of students in other grades. Thus, the country should follow up on the potential impact of COVID-19 on adolescents, such as the deterioration in mental health and economic status in adulthood. In a study conducted among adolescents aged 12–18 in China, depression and anxiety increased with grade level [24]. In addition, we observed that adolescents with low and high academic performance experienced suicidal ideation and loneliness more frequently than average performers. Adolescents in the low-performing group reported high levels of depression and unhappiness. Even before the COVID-19 outbreak, youth education played an important role in society owing to the cultural characteristics of Korea, and the stress produced by the highly competitive education system has negatively impacted the physical and mental health of adolescents [25]. Previous studies have reported a link between academic stress and depression [26, 27]. In a study investigating the risk factors related to suicide attempts among 106 depressed adolescents, 47.2% were classified into the suicide attempt group and depressive symptoms were found to be related to suicidal thoughts and suicide attempts [28]. Another study reported that implementing intervention programs according to adolescent grades could help reduce suicidal impulses [29].

Our study found that adolescents who perceived their household economic status as low had higher levels of depression, suicidal ideation, unhappiness, and loneliness. In addition, youths who had received financial support from others in the past 12 months had higher levels of depression, suicidal ideation, loneliness, and lower levels of unhappiness. A previous study using representative data from adolescents in Korea reported that low household economic status is associated with poor mental health [29]. A study of American adolescents reported that 'perceived financial stress' and 'family conflict' had a significant relationship with mental health problems, and these factors were mediated by the relationship between family difficulties and mental health problems [30]. The Ministry of Education of the Republic of Korea provided online classes when in-person classes were restricted because of COVID-19 [31], but it has been a challenge for groups with low household income to prepare for such classes, as they require equipment such as smartphones and computers [32]. The abuse of smartphones is closely related to mental health issues [33], and the policy of online classes has deprived young people of many important physical and mental health-related opportunities that could have been obtained through student‒teacher interactions [2], indicating that COVID-19 has deepened inequality.

Our study found that levels of stress, loneliness, suicidal ideation, depression, and unhappiness were 1.14‒2.23 times higher in groups that had been economically affected by COVID-19 than in the unaffected group. A study in Japan reported that adolescents experiencing unhappiness exhibited poor health behaviors and have poor mental health [34]. In a meta-analysis investigating the connection between socioeconomic inequality and mental health problems among adolescents, poor mental health was found to be associated with low economic status [4], while another study reported that economically vulnerable groups had a high fatality rate from COVID-19 [35]. Problems such as social isolation and economic deterioration caused by COVID-19 have resulted in irregular workers losing their jobs worldwide, worsening the situation of the poor [36]. Previous studies have reported that parental unemployment and financial difficulties can negatively affect adolescents’ mental health and well-being [37, 38]. In another study, economic inequality and mental health problems manifested differently according to the family structure. Adolescents from single-parent and reconstituted families in Korea are at high risk of poor mental health [39]. Most of these families belong to low socioeconomic groups [40]. The poor mental health of these adolescents is likely to worsen as their treatment is discontinued because of financial hardships and lockdown measures during the COVID-19 pandemic [41, 42]. Previous studies have shown that differences in mental health problems among adolescents may affect their future families, income, and employment [39]. The high incidence of suicidal ideation in the group that experienced economic losses due to COVID-19 may lead to increased suicide rates. As suicidal ideation and planning are important predictors of suicide [43], appropriate monitoring and interventions are needed to prevent an increase in suicide rates due to prolonged COVID-19 measures. The incidence of COVID-19 is decreasing because of the development of vaccines and treatments [44, 45]; however, problems associated with the after-effects of the disease are emerging [11]. Our findings can serve as a basis for formulating appropriate policies and government measures to protect Korean youth from the potential side-effects of the COVID-19 pandemic that could impair the rest of their lives. Effective delivery of knowledge has been confirmed as a factor in alleviating anxiety among teenagers during the COVID-19 pandemic [46], and art therapy was helpful for individuals during the Ebola epidemic [47]. Additional intervention in family conflicts is needed, and protective factors for the relationship between the economic impact of COVID-19 and poor mental health of adolescents in the future need to be identified.

Our study had some limitations. First, because this was a cross-sectional study, causal inferences regarding COVID-19 cannot be drawn. The potential impact of COVID-19 on adolescent mental health should be investigated in depth by monitoring and collecting data on their health behaviors. Second, because this study used secondary data collected by the state, we could not use a standardized questionnaire to specifically measure mental health problems, and factors such as social distancing, which could negatively affect mental health, were not adjusted for in the analysis. However, the data used in this study are relevant to understanding the current state of mental health during the pandemic, as we used data from Korean adolescents. Third, because the web survey was based on adolescents' self-awareness, their responses may have been biased. Specifically, perceived family economic status did not reflect household income. The strength of our study is that we used nationally representative probability-based samples at the same time, enabling us to estimate and understand the link between the economic damage caused by COVID-19 and mental health problems.

## Conclusions

The findings revealed an association between the decline in household economic status due to COVID-19 and mental health problems, such as stress, loneliness, suicidal ideation, depression, and unhappiness. Thus, COVID-19 may have accelerated economic and mental health inequalities among adolescents. Our findings can serve as a basis for formulating appropriate policies and government measures to protect Korean youth from the potential side-effects of the COVID-19 pandemic that could impair the rest of their lives. This result also strengthens the relationship between financial burden and poor mental health as perceived by adolescents. We hope that these findings will help improve the mental health of adolescents worldwide.

## Supplementary Information

**Additional file 1: Appendix 1.** General characteristics by depression and suicide ideation in the KYRBS 2020 adolescents. **Appendix 2.** General characteristics by unhappiness, lonely and stress in the KYRBS 2020 adolescents.

## Data Availability

The datasets used in this study are available from the corresponding author upon request. The KYRBS data can be accessed and downloaded from the KDCA website (https://www.kdca.go.kr/yhs/home.jsp).

## Abbreviations

COVID-19: The coronavirus disease-19
KYRBS: Korean Youth Risk Behavior Web-based Survey
KDCA: The Korea Disease Control and Prevention Agency
ORs: Odds ratios
CI: 95%Confidence interval
