# Supplementary material for: The association mental health of adolescents with economic impact during the COVID-19 pandemic: a 2020 Korean nationally representative survey
Source: BMC Public Health. 2023 May 11;23:853. doi: 10.1186/s12889-023-15808-3 (PMC10172067; doi:10.1186/s12889-023-15808-3)
Supplement: Supplementary file 1 — Additional file 1: Appendix 1. General characteristics by depression and suicide ideation in the KYRBS 2020 adolescents. Appendix 2. General characteristics by unhappiness, lonely and stress in the KYRBS 2020 adolescents. [file 12889_2023_15808_MOESM1_ESM.docx]

**Appendix 1. General characteristics by depression and suicide ideation in the KYRBS 2020 adolescents**

| **Variables** | **Participants** | **Depression** | | | **Suicide ideation** | | |
| --- | --- | --- | --- | --- | --- | --- | --- |
|  | **N(%)** | **Yes** | **No** | ***p-value*** | **Yes** | **No** | ***p-value*** |
| **Total** | 54,948 | 13,840 | 41,108 |  | 5,979 | 48,969 |  |
| **Age(median)** | 15(14-17) |  |  |  |  |  |  |
| **Gender** |  |  |  | <.001 |  |  | <.001 |
| Boys | 28,353(51.6) | 5,633 | 22,720 |  | 2,254 | 26,099 |  |
| Girls | 26,595(48.4) | 8,207 | 18,388 |  | 3,725 | 22,870 |  |
| **School grade(14-19years)** |  |  |  | <.001 |  |  | <.001 |
| Middle school 1^st^ | 10,005(18.2) | 2,030 | 7,975 |  | 897 | 9,108 |  |
| Middle school 2^nd^ | 9,564(17.1) | 2,281 | 7,283 |  | 1,063 | 8,501 |  |
| Middle school 3^rd^ | 9,392(17.1) | 2,429 | 6,963 |  | 1,053 | 8,339 |  |
| High school 1^st^ | 8,907(16.2) | 2,244 | 6,663 |  | 926 | 7,981 |  |
| High school 2^nd^ | 8,907(16.2) | 2,476 | 6,431 |  | 1,085 | 7,822 |  |
| High school 3^rd^ | 8,173(14.9) | 2,380 | 5,793 |  | 955 | 7,218 |  |
| **Perceived academic achievement** |  |  |  | <.001 |  |  | <.001 |
| High | 6,736(12.3) | 1,424 | 5,312 |  | 655 | 6,081 |  |
| Middle-High | 13,410(24.4) | 2,982 | 10,428 |  | 1,287 | 12,123 |  |
| Middle | 16,585(30.2) | 3,890 | 12,695 |  | 1,551 | 15,034 |  |
| Middle-Low | 12,684(23.1) | 3,589 | 9,095 |  | 1,534 | 11,150 |  |
| Low | 5,533(10.1) | 1,955 | 3,578 |  | 952 | 4,581 |  |
| **Perceived family economic status** |  |  |  | <.001 |  |  | <.001 |
| High | 6,039(11.2) | 1,350 | 4,689 |  | 521 | 5,518 |  |
| Middle-High | 15,300(28.6) | 3,576 | 11,724 |  | 1,487 | 13,813 |  |
| Middle | 26,397(47.5) | 6,385 | 20,012 |  | 2,639 | 23,758 |  |
| Middle-Low | 5,937(10.4) | 1,972 | 3,965 |  | 1,022 | 4,915 |  |
| Low | 1,275(2.2) | 557 | 718 |  | 310 | 965 |  |
| **Economic support**  **(in the last 12 months)** |  |  |  | <.001 |  |  | <.001 |
| No | 49,385(89.9) | 12,014 | 37,371 |  | 5,087 | 44,298 |  |
| Yes | 5,563(10.1) | 1,826 | 3,737 |  | 892 | 4,671 |  |
| **Economic impact of COVID-19** |  |  |  | <.001 |  |  | <.001 |
| No | 38,109(69.4) | 8,610 | 29,499 |  | 3,594 | 34,515 |  |
| Yes | 16,839(30.6) | 5,230 | 11,609 |  | 2,385 | 14,454 |  |

**Appendix 2. General characteristics by unhappiness, lonely and stress in the KYRBS 2020 adolescents**

| **Variables** | **Participants** | **Unhappiness** | **Lonely** | ***Stress*** |
| --- | --- | --- | --- | --- |
|  | **N(%)** | **Average** | **Average** | **Average** |
| **Total** | 54,948 | 2.19 | 2.41 | 3.17 |
| **Age(median)** | 15(14-17) |  |  |  |
| **Gender** |  |  |  |  |
| Boys | 28,353(51.6) | 2.08 | 2.22 | 3.01 |
| Girls | 26,595(48.4) | 2.30 | 2.61 | 3.34 |
| **School grade(14-19years)** |  |  |  |  |
| Middle school 1^st^ | 10,005(18.2) | 2.02 | 2.27 | 3.03 |
| Middle school 2^nd^ | 9,564(17.1) | 2.15 | 2.36 | 3.11 |
| Middle school 3^rd^ | 9,392(17.1) | 2.18 | 2.41 | 3.16 |
| High school 1^st^ | 8,907(16.2) | 2.20 | 2.46 | 3.17 |
| High school 2^nd^ | 8,907(16.2) | 2.29 | 2.50 | 3.26 |
| High school 3^rd^ | 8,173(14.9) | 2.31 | 2.47 | 3.33 |
| **Perceived academic achievement** |  |  |  |  |
| High | 6,736(12.3) | 1.96 | 2.31 | 3.07 |
| Middle-High | 13,410(24.4) | 2.07 | 2.37 | 3.10 |
| Middle | 16,585(30.2) | 2.16 | 2.35 | 3.14 |
| Middle-Low | 12,684(23.1) | 2.31 | 2.49 | 3.25 |
| Low | 5,533(10.1) | 2.52 | 2.60 | 3.38 |
| **Perceived family economic status** |  |  |  |  |
| High | 6,039(11.2) | 1.84 | 2.14 | 2.96 |
| Middle-High | 15,300(28.6) | 2.04 | 2.35 | 3.11 |
| Middle | 26,397(47.5) | 2.24 | 2.40 | 3.17 |
| Middle-Low | 5,937(10.4) | 2.53 | 2.74 | 3.42 |
| Low | 1,275(2.2) | 2.75 | 2.86 | 3.61 |
| **Economic support**  **(in the last 12 months)** |  |  |  |  |
| No | 49,385(89.9) | 2.17 | 2.38 | 3.15 |
| Yes | 5,563(10.1) | 2.32 | 2.65 | 3.33 |
| **Economic impact of COVID-19** |  |  |  |  |
| No | 38,109(69.4) | 2.13 | 2.33 | 3.11 |
| Yes | 16,839(30.6) | 2.31 | 2.59 | 3.31 |
